# Prevalence and morphometric analysis of the retromolar canal in a Spanish population sample: a helical CT scan study

**DOI:** 10.4317/medoral.25069

**Published:** 2021-10-27

**Authors:** Miguel Puche-Roses, Arantxa Blasco-Serra, Alfonso A Valverde-Navarro, Miguel Puche-Torres

**Affiliations:** 1 Department of Human Anatomy and Embryology, University of Valencia, Valencia, Spain; 2 Head of Department of Maxillofacial Surgery, Hospital Clínico Universitario de Valencia, INCLIVA, Spain. Associate Professor, Department of Surgery, University of Valencia, Valencia, Spain. ORCID ID: 0000-0002-9833-0707. Scopus Author ID: 23477898100

## Abstract

**Background:**

The retromolar canal (RMC) is an anatomical variation of the mandibular canal (MC) whose identification and study should be considered given its implication in the surgical procedures of the retromolar area. The prevalence of the RMC widely varies according to previous studies and may be influenced by the followed study method. This work aimed to evaluate the prevalence of the RMC in a Spanish population sample.

**Material and Methods:**

For this purpose, 225 CT scan images (with a higher resolution than the cone beam CT used in other previous studies) from the Hospital Clínico Universitario de Valencia were analyzed. The Osirix MD® radiological image analysis system was applied to analyse the dimensions, location in the retromolar area and morphologic characteristics of the RMC by classifying them according to their typology. Furthermore, the relations between the RMC and gender, age and laterality were studied.

**Results:**

RMC prevalence was 23.1%. No significant relation between the presence of the canal and gender, age or laterality was found. Type Ia was the commonest type with a prevalence of 40.8%.

**Conclusions:**

Based on the results of this study, the RMC should be considered a frequent anatomical variation whose complete study is very important in daily clinical practice.

** Key words:**Retromolar canal, retromolar foramen, retromolar triangle, mandibular canal, mandibular nerve, inferior alveolar nerve, CT scan, Helical scan.

## Introduction

**Figure 1:**
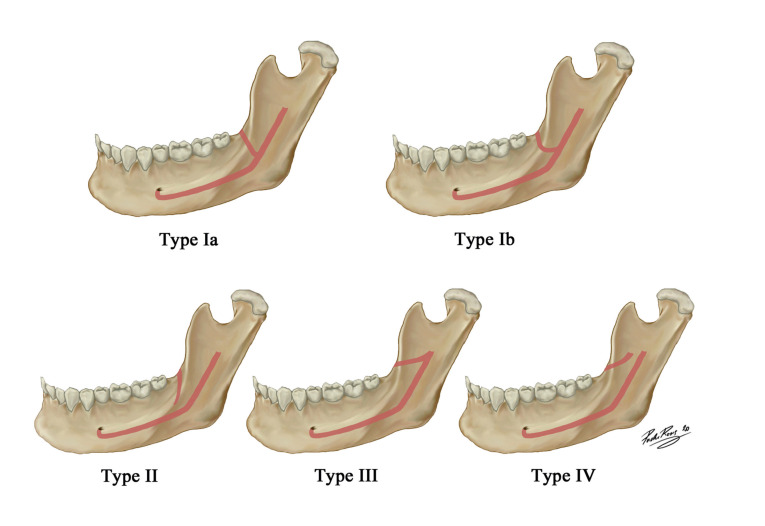
Illustration of RMC types: Type Ia originates from the MC and extends straight to the RMT; Type Ib originates from the MC and moves in a curved trajectory to the RMT; Type II, originates in the RMT and moves toward the root portion of the third molar with no connection to the MC; Type III originates from the MF and extends forward to the RMT; Type IV originates from a foramen other than the MF and extends anteriorly towards the RMT.

The retromolar canal (RMC) is an anatomical variation of the mandibular canal (MC) that originates from it and extends to its opening across the retromolar foramen (RMF) in the area of the retromolar trigone (RMT). It was described for the first time by Löfgren in 1957 and the first detailed analysis was published by Schejtman *et al*. in 1967. Since then, several works have evaluated the presence of the canal in different study populations. Some authors consider the RMC to be one of the anatomical variations of the MC, such as the bifid mandibular canal (BMC) (1). Others conversely classify the canal as a BMC type (2,3). Nevertheless, the present study considers this to be a relevant variation given the existence of different RMC types according to their origin, path and location. In 1987, Ossenberg (4) established a RMC differentiation according to the path in three basic types based on the direction and location of its origin from the MC. Since then different classification models have been described for the RMC: Patil *et al*. (2013) (5), Luangchana *et al*. (2018) (6) or Zhang *et al*. (2018) (7). This work classifies the RMC based on the classification of N. Nikkerdar *et al*. (2020) (Fig. 1) by taking it as the most complete one and by differentiating five RMC types (8).

Anatomical variations pose a problem when predicting any technique or surgical procedure’s success. Certain histological studies have confirmed that the RMC contains a neurovascular bundle composed of a myelinated nerve and blood vessels (one arteriola or more, and one venula or more) (4). Thus knowing RMC prevalence is relevant because its presence can generate intraoperative complications and/or functional postoperative after effects.

RMC prevalence very much changes in previous studies and might stem from the different exploration methods followed, from varying RMC concepts, and is considered a relevant anatomical independent variation or a BMC subtype given the differences in the sample well by number, composition or genetic factors (9). The works that have studied its prevalence by analysing panoramic radiographs have detected minor prevalences than those obtained by cone-beam computed tomography (CBCT) (3,10). Differences in prevalences have also been observed in some studies according to the population groups that formed the sample: Asia (between 8.5% and 75.4%) (5,11) and Europe (from 16.12% to 71.9%) (12,13). The only study performed with a Spanish population sample is based on the analysis of CBCT images and took the RMC to be a BMC type. It gave a prevalence of 12% (3). After the bibliographical review, we found that no works had studied either the prevalence, or the anatomical and morphometric characteristics, of the RMC by analysing helical CT scan images, even though this is one of the most widely used radiological skills to study the head and neck. Current image storage systems permit sufficient studies to be done of CT scans of hospiTable images stored in databases, which enables retrospective studies of this type to be conducted without having to irradiate patients.

The main aim of this work was to determine the prevalence and anatomical and morphometric characteristics of the RMC by analysing radiological images obtained by helical CT scans in a aleatory sample of adult patients from the Hospital Clínico Universitario de Valencia (HCUV) (east Spain).

## Material and Methods

This retrospective, quantitative and descriptive study was carried out at the HCUV and the Faculty of Medicine and Odontology of the University of Valencia.

The sample was selected from the radiological CT studies database stored in the Picture Archiving and Communication System (PACS) of the HCUV. The sample was formed by patients undergoing high-resolution craniofacial helical CT scans. The studies of patients older than 18 years old who were explored between 01-09-2019 and 01-09-2020 were selected. The following were taken as the exclusion criteria: presence of a medical history of traumatic, malformative, surgical or oncologic disease in the jaw and/or base of the skull; the CT scan study did not meet the quality criteria of the images; images presented artefacts.

All the CT scan studies were performed by the HCUV Radiodiagnostic Service with a multidetector CT scanner (MDCT), namely a Toshiba Aquilion™ Prime de 80 detector.

This study was approved by the Ethics Committee of Clinic Research of the HCUV, and by the Scientific Committee of the INCLIVA Health Research Institute of the HCUV (no.: 2020/337). To comply with Spanish Organic Law 3/2018, of 5 December, on Personal Data Protection and Guaranteeing Digital Rights, and to align with Law 41/2002 that regulates Patients’ Autonomy and Their Rights and Obligations, dissociative data processing was applied that consisted in the pseudonymisation of the sex and age data of those cases meeting the inclusion criteria. The selected images were stored in a digital imaging and communication on medicine (DICOM) format on a hard disk protected by a password to be analysed with no identification data shown on the screen (anonymisation).

The OsirixMD® (opencode software, 32 bits) radiological picture analysis system was used for the measurement procedure. The study was initially imported to the DICOM format. Then multiplanar reconstruction (MPR) was applied to detect the presence or absence of the RMC.

As soon as the presence of the RMC was confirmed, it was classified according to the typology described by N. Nikkerdar (2020) (8) (Fig. 1). Later RMC length (L) was measured by considering the distance in mm from its origin to its exit through the RMF. Its diameter (D) was calculated by using the mean of three measurements taken along the RMC path: 1/3 distal, 1/3 intermediate and 1/3 proximal.

**Figure 2:**
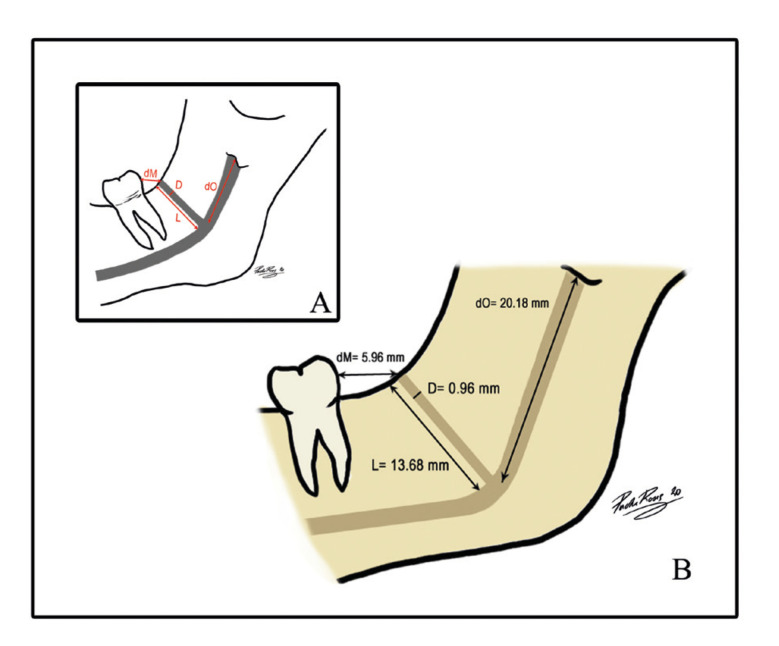
A.- Schematic drawing of morphometric measurements: (L) RMC length in mm, (D) RMC diameter in mm, (dO) Distance in mm from MF to the RMC origin, (dM) Distance in mm from the RMF to the third molar. B.- Illustration of the RMC archetype: a commoner type, and the mean dimensions and distances.

In order to evaluate the location of the RMC, the distance from the RMF to the crown of the third molar (dM) was measured in mm. In those cases in which the third molar was absent, a perpendicular line was traced from the most posterior maxillary tuberosity portion (the region where the third molar is normally found) and the distance to the RMF was measured in mm. Finally, the distance from the mandibular foramen (MF) (at the lingula or spine level of Spix) to the origin of the RMC (dO) was measured in mm (Fig. 2). This measurement cannot be calculated in RMC types II and IV because they do not communicate with the MC.

Statistical calculations were performed with the SPSS® v.26 (IBM) software. Firstly, by the chi-square test, RMC prevalence and its possible statistical relation with the sex, age and laterality variables was analysed. The variable age was stratified according to the different clinical profiles in four main age groups: 18-29; 30-49; 50-69, and ≥70 years.

The number of cases, means, standard deviations, and the maximum and minimal values, were described for the variables of dimension (L and D) and location (dO and dM) of the RMC. Normal data distribution was studied by the Kolmogorov-Smirnov test. Later measurements were compared according to RMC type by a one-factor ANOVA test (F) with Bonferroni’s post hoc test (or its non-parametric Kruskal-Wallis (H) equivalent with a later pairwise comparison by pairs by the Mann-Whitney U test in those cases in which the normality assumption was not fulfilled). Measurements were also compared according to sex by the Student’s t-test for the independent samples (or its equivalent non-parametric Mann-Whitney U test). The significance level was set at *p*<0.05 in all cases.

## Results

Following the aforementioned selection criteria, and after searching with the GIMD Visor Ligero® programme, 311 patients with a craneo-facial helical CT scan were obtained. Eighty-six patients were excluded from the study for being duplicated (8 cases), for bad quality images (26 cases) or for presenting jaw pathologies (52 cases, who 10 presented cysts at the RMT level, 8 traumatisms, 14 oncology pathologies, 3 malformative problems and 17 osteonecrosis cases). The final sample consisted in 225 adult patients, 86 men (38.2%) and 139 women (61.8%) aged between 18 and 94 years old (mean of 43.48±19 years).

The mean age of the male patients, which ranged from 18 to 90 years, was 46.03±19 years. The mean age of the women, whose ages went from 18 to 94 years, was 41.91±18.9 years. The distribution of the 225 patients in the four age groups was as follows: 67 (29.8%) in the 18-29-year-old group, 72 (32%) aged between 30-49 years, 64 (28.4%) between 50-69 years old and 22 (9.8%) in the ≥70-year-old group (Table 1).

After analysing the 225 CT scans images, 52 patients presented RMC anatomical variation, whose prevalence was 23.1%. Of the 52 individuals with RMC, 44.2% (23 patients) were men and 55.8% were women (29 patients). Of all the men (86 patients), the RMC was found in 26.7%, and in 20.9% of all the women (139 patients) (Table 2). No statistically significant relation was observed between the presence of RMC and patients’ sex (χ2=1.034, *p*=0.309).

The mean age of the individuals with an RMC was 38.4 years old with a standard deviation of 15.7 years. Groups were distributed as follows: 38.5% (20 individuals) in the 18-29-year-old group, 30.8% (16 individuals) aged between 30-49 years, 28.8% (15 individuals) between 50 and 69 years and 1.9% (1 individual) in the ≥70-year-old group (Table 2). As we can see, the prevalence of the individuals with RMC lowered with ageing. Nevertheless, the relation between the presence of RMC and age was not statistically significant (χ2=6.016, *p*=0.111).

Regarding laterality, of the 52 individuals with RMC, it was bilateral in 46.2% (24 patients), and was only unilateral in 53.8% (28 patients). With a unilateral RMC, 60.7% (17 canals) were on the right side and 39.3% (11 canals) on the left. The possible relation between sex and RMC laterality was evaluated and no statistically significant relation was found (χ2= 0.601, *p*=0.438) (Table 3).

**Figure 3:**
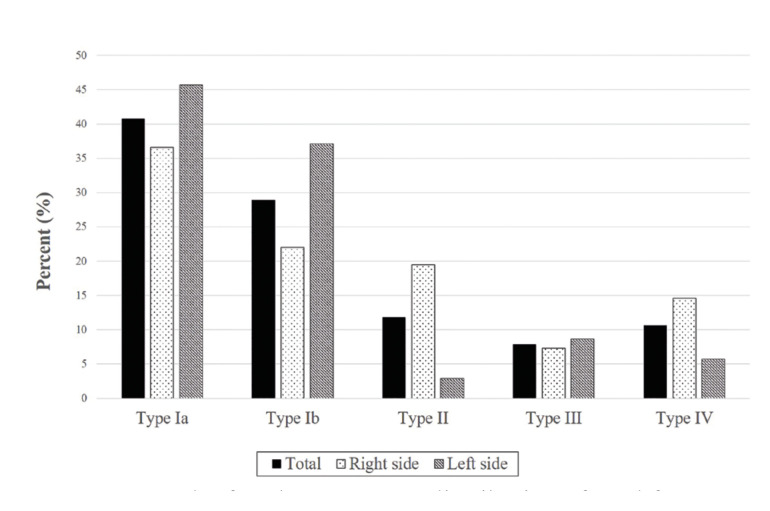
Bar graph of each RMC type, distribution of total frequency and according to the right and left side.

**Figure 4:**
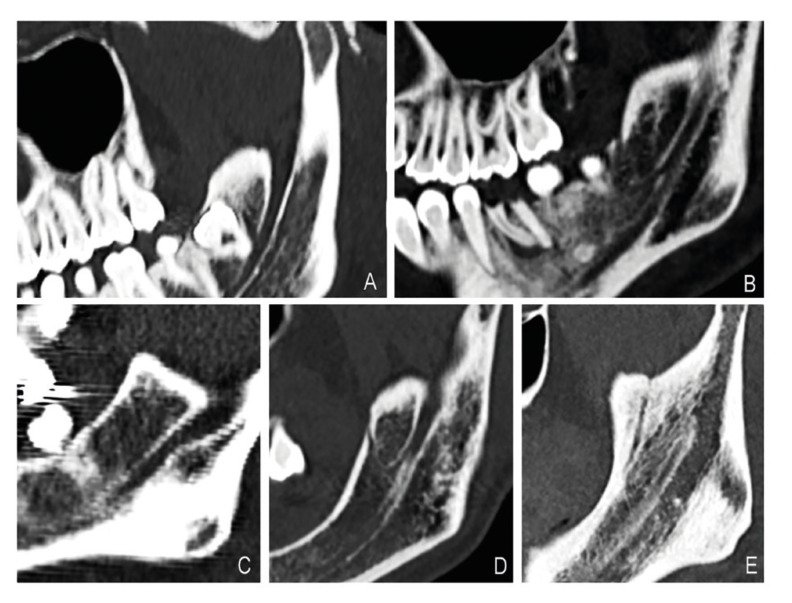
The RMC classification on the helical CT scan pictures found in this study. A: Type Ia; B: Type Ib; C: Type II; D: Type III; E: Type IV.

By classifying all the 76 found RMC, we observed that 31 canals belonged to type Ia, which was the commonest type with a prevalence of 40.8%. The prevalence of the other types was as follows: 22 canals (28.9%) of type Ib, 9 (11.8%) of type II, 6 (7.9%) of type III and 8 (10.6%) of type IV (Fig. 3, Fig. 4). The distribution of the prevalence of the different RMC types as unilateral or bilateral appears in Table 3. The possible relation between laterality and RMC type was evaluated and no statistically significant relation was found (χ2= 2.592 and *p*=0.628 on the right side; χ2= 5.605 and *p*=0.231 on the left side).

**Table 1:**
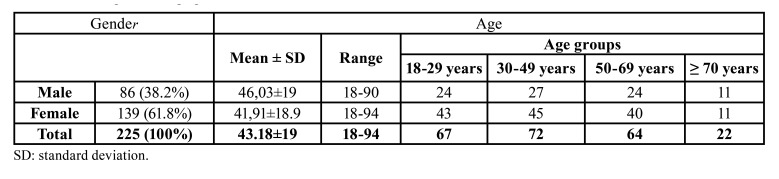
The sample’s demographic characteristics.

**Table 2:**
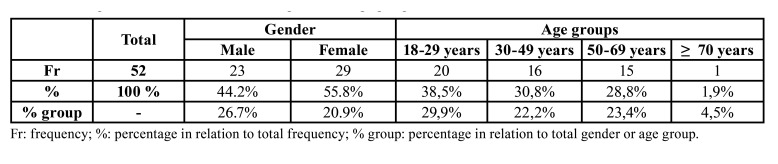
RMC prevalence distribution between gender and age groups.

**Table 3:**
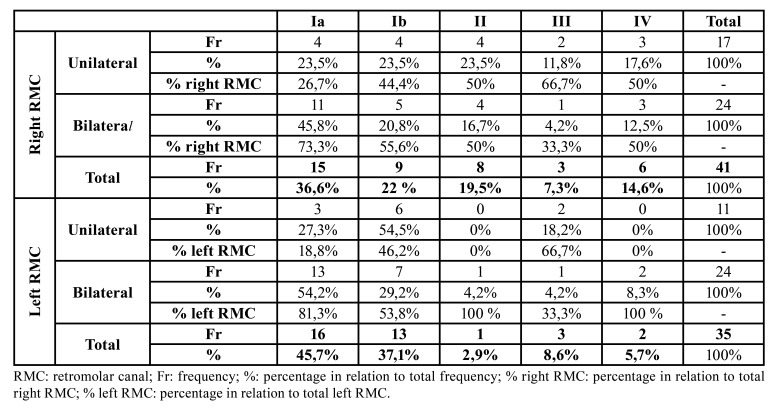
RMC distribution of types according to sides based on laterality (unilateral/bilateral).

The mean RMC length was 13.65±3.9 mm on the right side and it was 14.15±4.34 mm, with 13.05±3.28 mm, on the left side. The relation between length and canal type was analysed (Table 4). Statistically significant differences were found in length between types (H=18.741, *p*=0.001). These differences appeared between type IV canals, whose mean length was 20.65±5.23 mm, and type Ia canals (*p*=0.000), Ib (*p*=0.002) and II (*p*=0.09). Differences were observed between type Ia and type III (*p*=0.017). The differences in length for men showed a mean length of 13.96 mm and one of 13.37 mm for women. No statistically significant differences were found between length and patients’ sex (*p*=0.778).

The mean RMC diameter was 0.96±0.29 mm. On the right side, the mean diameter was 0.94±0.27 mm, and was 0.97±0.29 mm on the left side. Diameter was analysed according to RMC type (Table 4). Statistically significant differences appeared in the diameters between the different types (H=10.496, *p*=0.033). These differences were found between type Ia and type Ib (*p*=0.04), and between type II and types Ib (*p*=0.007), III (*p*=0.031) and IV (*p*=0.043). As for differences according to the sex, the mean diameter in men was 1.03 mm and 0.89 mm in women. These differences were not statistically significant (*p*=0.367).

The mean dM distance was 5.96±2.98 mm. No statistically significant differences were found between dM distance and the different canal types (H= 5.806, *p*= 0.214). The mean dM distance in men was 5.53±2.16 mm and 6.32±3.52 mm in women, which were not statistically significant (*p*=0.719).

**Table 4:**
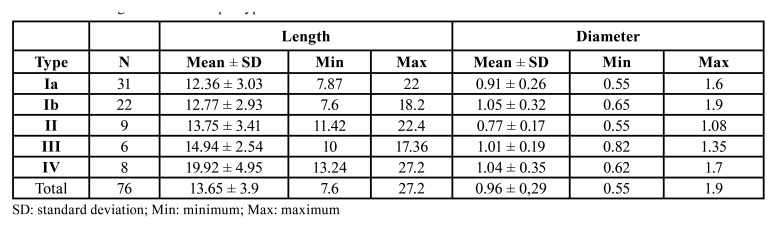
CRM length and diameter per type.

The mean dO distance was 20.18±7.04 mm. dO distance was analysed according to canal type by bearing in mind that this measurement was only applicable to the types that were connected to the MC (types Ia, Ib and III). Statistically significant differences between dO distance and types Ia and III were found (*p*=0.005). The mean dO distance in men was 23.1±6.25 mm and 17.88±6.85 mm in women. These differences were statistically significant (t=3.015, *p*=0.004). An RMC archetype (more prevalent) was defined based on the means of the measurements taken of both dimension (L and D) and location (dM and dO) (Fig. 2).

## Discussion

The RMC presents a few anatomical and morphometric characteristics that allow us to consider it to be a relevant variation in the MC in planning clinical surgical procedures of the retromolar area. Knowing its presence will allow us to avoid after effects, such as anaesthetic block failures (14) or damage to neurovascular bundle, post-surgery haemorrhaging and hypoesthesias (15) of the third molar (12), sagital split osteotomy during orthognatic surgery (16) or autologous bone harvesting in the RMT (17).

This study evaluated the prevalence of the RMC in a sample of 225 patients, of whom 38.2% were men and 61.8% were women, which are similar Figures to those reported in previous studies (3,6). RMC prevalence vastly changes according to different studies from 0% as observed by Kikuta *et al*. (2018) (18) by studying panoramic radiographs to 75.4% as noted in the CBCT images by Patil *et al*. (2013) (5). In our study, the observed prevalence was 23.1%, which coincides with other studies based on CBCT with similar sample sizes (8,19). There may be several causes for the different prevalence indicated by several studies, such as the applied anatomical definition of the RMC, different sample sizes in studies, samples’ distinct population origins, study designs, e.g. with patients or cadaveric mandibles, varying inclusion and exclusion criteria, the applied radiological assessment method or interpersonal variability in researchers’ detection abilities.

The results of the present study showed no significant differences between males and females, which falls in line with other published studies (4,5,6,19,20). Only Akhtar *et al*. (2014) (10) found that the RMC was more frequent in females.

Regarding the age profile, the RMC was commoner in the 18-29 year-old age group. This finding agrees with the study by Ossenberg (1987) (4), who reported a peak incidence in an adolescent cohort. No significant differences were found among age groups, which is consistent with the results of von Arx *et al*. (2011) (21). However, we found that the RMC prevalence lowered with ageing. One of the reasons for this could be the need for greater neurovascular contribution as regards thirds molars emerging in young people. Furthermore, lower prevalences in older people could be due to remodelling, loss of bone density and the higher prevalence of toothless patients.

In the present study, although the unilateral RMC was more prevalent, this difference was not significant. This finding has often been reported in other studies (4,21,22), and the unilateral percentage is similar to that indicted in the study of Patil *et al*. (2013) (55.5%) (5). According to the unilateral canals, we found that right canals were more frequent than left canals, just as Nikkerdar *et al*. reported, but with no significant differences (8). Nevertheless, higher prevalences have been described for left canals (5,21).

The commonest RCM type in this study was type Ia, which agrees with the results of both von Arx *et al*. (2011) (21) and Filo *et al*. (2015) (12). Ib was the second commonest type, which makes type I the most prevalent type canal (69.7%) and agrees with that reported by Sisman *et al*. (2015) (23). Concerning typology, we had to consider the diversity of the classifications used in all the different studies to explain their disparity. Some classifications do not include types II and IV because they do not communicate with the MC. This fact also comes over in those works that consider the RMC to be a BMC type, which could imply underestimating the real RMC prevalence, and it could be worse if we bear in mind that some studies have described type II as the most prevalent RMC type (5,6,8).

The present study analysed RMC dimensions using length and diameter measurements. The mean RMC length was 13.65 mm by measuring from its origin to the RMF. These length measurements are not comparable to other previous studies because they do not evaluate real RMC length. Previous studies have measured height (vertical distance) and what they call ‘length’ (anteroposterior distance). In this way, they establish a location of the canal in the mandible, but did not study its real dimensions (3,23,24,25).

Our study found significant differences between RMC type IV length and types Ia, Ib and II. This finding makes sense because type IV canals are the so-called temporal crest canals, which arise in this mandibular area and have to cross a marked stretch to reach the retromolar fossa. The same applies to the differences found between type III and type Ia.

The mean RMC diameter was 0.96 mm, which comes very close to the results of other studies (12,21) and within the range between reported minor (0.27 mm) and major (3.29 mm) diameters (25). Diameters of canals could also be determined by canal type as it has been observed that type II canals have a diameter of 0.5 mm or smaller (8). This fact came over in our study given the differences in the diameter between type II and types Ib, III and IV.

Otherwise, the RMC location was evaluated by measuring the distance from the RMF to the third molar, with a mean of 5.96 mm, which comes closer to that described by Park *et al*. in 2016 (5.8 mm) (24). This mean distance falls within the range reported in the literature: 4.23 mm (22) and 7.1 mm (5). The other measurement for location was the distance from the MF to the RMC origin, with a mean of 20.18 mm in this study, which is similar to the distance reported by Park *et al*. in 2016 (21.5 mm) (24).

Another of the reasons proposed as a cause of the differences in prevalence among several studies is the influence of population origins. The first to describe this was Ossenberg (4), who observed that the presence of RMF was more frequent in individuals from North America than from other populations from Europe, India, NE Asia and Africa. Nevertheless, other studies have found no differences in the RMF prevalence in the jaws of white and black individuals (20). High prevalences have also been described in works done with a Japanese population (75.4%) (5) and a European population (71.9%) (13).

In the same ethnic group, we found some studies with very varied prevalences. In a Japanese population, prevalences went from 3.2% to 75.4%, along with other intermediate prevalences (4,5,18,26). An explanation for this could lie in the different study methods followed. We also found studies conducted in the same population that used the same radiological assessment method (CBCT), but presented very different prevalences; e.g. an Iranian population with prevalences between 7.3% and 25.4% (10,27).

In the only previous study performed with a Spanish population in our bibliographical review, the RMC prevalence of all the patients was 12% (3), which is lower than our prevalence of 23.1%. That former study also analysed a sample of 225 patients, but it took the RMC to be a BMC type and employed CBCT images, which could account for the difference in their prevalence compared to our work.

Undoubtedly, the most relevant factor when explaining differences among different interstudy prevalences is the employed radiological assessment method. This fact has been demonstrated in many studies showing the CBCT’s clear superiority as opposed to panoramic radiographs by detecting a high prevalence of using CBCT and demonstrating that panoramic radiographs did not detect some RMCs (3,18,21,23). This fact emphasises the importance of detecting the RMC with methods that involve more suiTable characteristics, with no overlapping of structures and with a higher resolution. Indeed the study of Patil *et al*. (2013) used CBCT with a 0.08 mm voxel size, i.e. high-resolution image, and found a prevalence that was 75.4%, higher than any other study. This might be partly due to the higher detection capacity, and indicates that high-resolution images could be very useful when detecting the RMC (5). It might also serve to better identify type II canals, whose diameters are smaller than the rest, which sometimes does not allow them to be detected (8).

The principal difference between the helical CT scan and CBCT lies in differentiating hard tissue from soft tissue simultaneously during the same examination, and presenting a better trabecular outline and a higher resolution (13). Hence using helical CT scan technology would lead us to more reliable prevalences, measurements and analyses of anatomical RMC, which come closer to reality. To date, this is the first study to analyse the prevalence and the anatomical and morphometric characteristics of the RMC by helical CT scan images. We found no works in our bibliographical review that have applied this technology, while the majority of works report CBCT images.

Another radiological method is magnetic resonance imaging (MRI), which has only been used in one work to study the inferior alveolar nerve and its divisions in six cadaveric heads to find retromolar branches (28). The inferior alveolar neurovascular bundle seems to be the commonest origin of RMC content (4). The large-scale visualisation of soft tissue makes MRI the best method for studying RMC content and its origins. As we found no known clinical importance of studying the RMC by MRI, more works in this research line would be interesting.

Lastly, the RMC has also been studied by a non-radiological assessment method: endoscopy. A study by Iwanaga *et al*. (2017) reported a prevalence of 18.8% by analysing 66 sides of mandible bones by a 2 mm endoscope (2 mm diameter) through the MC (26).

## Conclusions

Based on the prevalence found in this study, the RMC is considered a frequent anatomical variation. No relation with gender, age and side was found. Type Ia was the commonest RMC type. Finally, we defined an RMC archetype based on the summary of the most frequent anatomical and morphometric characteristics.

## References

[1] Haas LF, Dutra K, Porporatti AL, Mezzomo LA, De Luca Canto G, Flores-Mir C (2016). Anatomical variations of mandibular canal detected by panoramic radiography and CT: a systematic review and meta-analysis. Dentomaxillofacial Radiol.

[2] Naitoh M, Hiraiwa Y, Aimiya H, Ariji E (2009). Observation of bifid mandibular canal using cone-beam computerized tomography. Int J Oral Maxillofac Implants.

[3] Muinelo-Lorenzo J, Suárez-Quintanilla JA, Fernández-Alonso A, Marsillas-Rascado S, Suárez-Cunqueiro MM (2014). Descriptive study of the bifid mandibular canals and retromolar foramina: cone beam CT vs panoramic radiography. Dentomaxillofacial Radiol.

[4] Ossenberg NS (1987). Retromolar foramen of the human mandible. Am J Phys Anthropol.

[5] Patil S, Matsuda Y, Nakajima K, Araki K, Okano T (2013). Retromolar canals as observed on cone-beam computed tomography: their incidence, course, and characteristics. Oral Surg Oral Med Oral Pathol Oral Radiol.

[6] Luangchana P, Pornprasertsuk-Damrongsri S, Kitisubkanchana J, Wongchuensoontorn C (2018). The retromolar canal and its variations: Classification using cone beam computed tomography. Quintessence Int Berl Ger 1985.

[7] Zhang YQ, Zhao YN, Liu DG, Meng Y, Ma XC (2018). Bifid variations of the mandibular canal: cone beam computed tomography evaluation of 1000 Northern Chinese patients. Oral Surg Oral Med Oral Pathol Oral Radiol.

[8] Nikkerdar N, Golshah A, Norouzi M, Falah-Kooshki S (2020). Incidence and Anatomical Properties of Retromolar Canal in an Iranian Population: A Cone-Beam Computed Tomography Study. Int J Dent.

[9] Truong MK, He P, Adeeb N, Oskouian RJ, Tubbs RS, Iwanaga J (2017). Clinical Anatomy and Significance of the Retromolar Foramina and Their Canals: A Literature Review. Cureus.

[10] Ngeow WC, Chai WL (2021). The clinical significance of the retromolar canal and foramen in dentistry. Clin Anat N Y N.

[11] Han SS, Hwang YS (2014). Cone beam CT findings of retromolar canals in a Korean population. Surg Radiol Anat.

[12] Filo K, Schneider T, Kruse AL, Locher M, Grätz KW, Lübbers H T (2015). Frequency and anatomy of the retromolar canal - implications for the dental practice. Swiss Dent J.

[13] Moreno Rabie C, Vranckx M, Rusque MI, Deambrosi C, Ockerman A, Politis C (2019). Anatomical relation of third molars and the retromolar canal. Br J Oral Maxillofac Surg.

[14] Palti DG, Almeida CM de, Rodrigues A de C, Andreo JC, Lima JEO (2011). Anesthetic technique for inferior alveolar nerve block: a new approach. J Appl Oral Sci Rev FOB.

[15] Azaz B, Lustmann J (1973). Anatomical configurations in dry mandibles. Br J Oral Surg.

[16] Yamamoto R, Nakamura A, Ohno K, Michi K (2002). Relationship of the mandibular canal to the lateral cortex of the mandibular ramus as a factor in the development of neurosensory disturbance after bilateral sagittal split osteotomy. J Oral Maxillofac Surg.

[17] Khoury F, Hanser T (2015). Mandibular Bone Block Harvesting from the Retromolar Region: A 10-Year Prospective Clinical Study. Int J Oral Maxillofac Implants.

[18] Kikuta S, Iwanaga J, Nakamura K, Hino K, Nakamura M, Kusukawa J (2018). The retromolar canals and foramina: radiographic observation and application to oral surgery. Surg Radiol Anat.

[19] Lizio G, Pelliccioni GA, Ghigi G, Fanelli A, Marchetti C (2013). Radiographic assessment of the mandibular retromolar canal using cone-beam computed tomography. Acta Odontol Scand.

[20] Alves N, Deana NF (2015). Anatomical and radiographical study of the retromolar canal and retromolar foramen in macerated mandibles. Int J Clin Exp Med.

[21] von Arx T, Hänni A, Sendi P, Buser D, Bornstein MM (2011). Radiographic Study of the Mandibular Retromolar Canal: An Anatomic Structure with Clinical Importance. J Endod.

[22] Bilecenoglu B, Tuncer N (2006). Clinical and Anatomical Study of Retromolar Foramen and Canal. J Oral Maxillofac Surg.

[23] Sisman Y, Ercan-Sekerci A, Payveren-Arikan M, Sahman H (2015). Diagnostic accuracy of cone-beam CT compared with panoramic images in predicting retromolar canal during extraction of impacted mandibular third molars. Med Oral Patol Oral Cirugia Bucal.

[24] Park MK, Jung W, Bae JH, Kwak HH (2016). Anatomical and radiographic study of the mandibular retromolar canal. J Dent Sci.

[25] Kang JH, Lee KS, Oh MG, Choi HY, Lee SR, Oh SH (2014). The incidence and configuration of the bifid mandibular canal in Koreans by using cone-beam computed tomography. Imaging Sci Dent.

[26] Iwanaga J, Watanabe K, Saga T, Tubbs RS, Tanaka K, Kikuta S (2017). A Novel Method for Observation of the Mandibular Foramen: Application to a Better Understanding of Dental Anatomy. Anat Rec Hoboken NJ 2007.

[27] Afsa M, Rahmati H (2017). Branching of mandibular canal on cone beam computed tomography images. Singapore Dent J.

[28] Ikeda K, Ho KC, Nowicki BH, Haughton VM (1996). Multiplanar MR and anatomic study of the mandibular canal. Am J Neuroradiol.

